# Ego, drives, and the dynamics of internal objects

**DOI:** 10.3389/fpsyg.2014.00666

**Published:** 2014-07-01

**Authors:** Simon Boag

**Affiliations:** Department of Psychology, Macquarie UniversitySydney, NSW, Australia

**Keywords:** ego, instinctual drives, internal objects, metapsychology, object-relations, repression, self, structural theory

## Abstract

This paper addresses the relationship between the ego, id, and internal objects. While ego psychology views the ego as autonomous of the drives, a less well-known alternative position views the ego as constituted by the drives. Based on Freud’s ego-instinct account, this position has developed into a school of thought which postulates that the drives act as knowers. Given that there are multiple drives, this position proposes that personality is constituted by multiple knowers. Following on from Freud, the ego is viewed as a composite sub-set of the instinctual drives (ego-drives), whereas those drives cut off from expression form the id. The nature of the “self” is developed in terms of identification and the possibility of multiple personalities is also established. This account is then extended to object-relations and the explanatory value of the ego-drive account is discussed in terms of the addressing the nature of ego-structures and the dynamic nature of internal objects. Finally, the impact of psychological conflict and the significance of repression for understanding the nature of splits within the psyche are also discussed.

The precise relationship between the id, ego, and instinctual drives remains an issue of dispute. The most prominent post-Freudian position proposes that the ego is independent of the instinctual drives (e.g., Hartmann, 1950, 1958). However, an alternative, albeit less-recognized, school of thought proposes instead that it is the instinctual drives *as knowers* that constitute the ego. The first attempt to clearly articulate this position originates in the writings of Maze (1983, 1987, 1993) although antecedents can be seen in the writings of earlier authors (e.g., Passmore, 1935, p. 282). Following clues from Freud’s writings concerning the “ego-instincts” (e.g., Freud, 1910), Maze’s position postulates that the knowing subjects within the individual are the instinctual drives. Furthermore, given that there are *multiple* instinctual drives, each person then consists of a multiplicity of knowers. Consequently, this position has radical implications for conceptualizing the id, ego, and superego, and personality structures in general.

However, assessing any theory about the ego, id and superego is not straight-forward. While Freud’s position is based upon empirical observation, empirical evaluation of any theory also requires prior theoretical clarification. Accordingly, refining our understanding of psychoanalytic theory and concepts is essential for progress in psychoanalysis. An added obstacle here, though, is that psychoanalytic schools of thought are diverse (Wallerstein, 2005a, b), contributing to what is described as “theoretical chaos” (Green, 2005, p. 629). One possible common ground, however, is to return to Freud’s theory since a “very minimal version of Freudian theory is accepted by almost all who accept any version of psychoanalytic theory” (Erwin, 1988, p. 243). However, as is well-recognized, evaluating the complexity of Freudian theory is itself difficult, partly due to “unresolved contradictions in Freud’s writings” (Shill, 2004, p. 125). In fact, anyone systematically reading Freud will likely agree with Madison’s (1956) observation that Freud’s writings:

… represent an historical account of an adventurous explorer developing a system of concepts that changed and grew continuously and unevenly over a half-century of creative effort … [subsequently he] left behind a trail of complex ideas unevenly developed and never integrated into a logical, systematic whole (p. 75).

Despite this, the importance of theoretical clarification cannot be over-stated since theory provides the major explanatory foundation for understanding clinical findings and therapeutic success in psychoanalysis. For this reason, both conceptual and theoretical research is an indispensable tool for assessing psychoanalytic claims, both with respect to theoretical clarification and generating theoretical implications for empirical assessment (see Dreher, 2005; Leuzinger-Bohleber and Fischmann, 2006; Brakel, 2009, 2013; Boag, 2012).

Using theoretical and conceptual analysis, the broad aims of this paper are to contribute theoretical clarification and extension to the accounts of the id, ego, and superego and to then provide a synthesis of this account with object relations accounts that postulate multiple ego-structures and dynamic internal objects. More specifically, the paper aims to: (a) develop novel theoretical insights by assessing the logical implications of an ego-drive account, and; (b) synthesize these theoretical findings with object-relation approaches to address the dynamics of internal objects. To achieve this, this paper first discusses the “ego” concept in psychoanalytic thinking and its relationship to the id. After discussing problems with the view that the ego is autonomous of the id, a theory of ego-drive theory is then advanced. On this viewpoint, a sub-set of the instinctual drives directly compose the ego and this sub-set can be further divided to account for the superego (intra-ego conflict). This position is then applied to a general theory of ego-differentiation and ego-structures, as well as justifying the dynamics of internal objects in object-relations approaches.

## THE EGO AND THE ID IN FREUDIAN THINKING

The ego (*Ich*) and id (*Es*) first formally appear in 1923 although antecedents are found in both Freud’s *Project* (Freud, 1895) and his earlier topographic theory (e.g., Freud, 1900). It was short-comings of the latter which prompted Freud to revise the conscious-unconscious relationship and postulate a revised model of personality that has come to be known as the “structural” theory. These structures consist of the ego, id and superego and according to Freud the id and ego can be understood in terms of how they differ from one another. For instance, consciousness is attached to the ego whereas the id is unconscious; the ego is that which knows and that which can be known (even if some aspects of ego functioning such as defences are unconscious – Freud, 1923b, pp. 17–18), while the id consists of “the dark, inaccessible part of our personality” (Freud, 1933, p. 73). Furthermore, the ego is structured, organized, and possesses a synthetic character which is uncharacteristic of the id: “what distinguishes the ego from the id quite especially is a tendency to synthesis in its contents, to a combination and unification in its mental processes which are totally lacking in the id” (Freud, 1933, p. 76). The ego is also an *agency* that controls and initiates action whereas the id can only act through influencing the ego:

… in each individual there is a coherent organization of mental processes; and we call this his *ego*. It is to this ego that consciousness is attached; the ego controls the approaches to motility… it is the mental agency which supervises all its own constituent processes….

Freud, 1923b, p. 17, his italics

However, the id is further conceptualized in two (not mutually exclusive) ways: one as the biological unconscious instinctual drives; the other as that which is repressed. With the former, the id is “a cauldron full of seething excitations. We picture it as being open at its end to somatic influences, and as there taking up into itself instinctual needs which find their psychical expression in it” (Freud, 1933, p. 73). This id is primarily concerned with gratification, without regard to external constraints or possible consequences and, unlike the ego, is only sensitive to internal stimulation and “has no direct communication with the external world” (Freud, 1940, p. 197). The other component of the id is that of the repressed. Freud (1923b) writes that “the repressed merges into the id as well, and is merely a part of it” (p. 24; cf. Freud, 1933, p. 77). As the repressed, the id consists of all those impulses subjected to repression, which remain unaffected by time and which partake in the particular processes of the biological id (Freud, 1933, p. 74).

The processes that the id and ego differ on reflect Freud’s primary *Ucs*. processes and *Cs*./*Pcs.* secondary processes distinction, and Gill (1963) writes that “the criteria of *Ucs*. and *Pcs*. are the same as those of id and ego” (p. 53; cf. Compton, 1972, 1981; Wiedeman, 1972; Wollheim, 1991; Brenner, 1994; Boesky, 1995; Petocz, 1999). The id here is described as operating via the primary process *pleasure* principle whereas the ego is motivated by the *reality* principle: “Just as the id is directed exclusively to obtaining pleasure, so the ego is governed by considerations of safety. The ego has set itself the task of self-preservation, which the id appears to neglect” (Freud, 1940, p. 199; cf. Freud, 1923b, p. 56).

Due to the id’s lack of concern for external reality and safety, the ego assumes the role of an *executive agent*, attempting to satisfy the id through activity in the world: “As a frontier-creature, the ego tries to mediate between the world and the id, to make the id pliable to the world and, by means of its muscular activity, to make the world fall in with the wishes of the id” (Freud, 1923b, p. 56; cf. Freud, 1924, p. 167, Freud, 1933, p. 75). Accordingly, the ego is viewed as a regulating agent in charge of balancing the demands of the “irrational” id, super-ego, and constraints of the external world (Freud, 1923b, p. 55; Freud, 1940, p. 199). The ego functions here via cognitive activity and perception, anticipating danger, and both preparing responses and inhibiting action (Freud, 1940, p. 199).

## PROBLEMS WITH THE EGO AND THE ID

Freud’s account generally spells out the id and ego in terms of their functions (i.e., what they are said to do). A problem with such functional definitions is that it is not entirely clear *what* it is that is said to be performing these functions and such definitions lend themselves to reification and circular explanation whereby a set of processes is treated as an entity performing those same processes (and then used to explain those same processes with respect to the concocted entity – see Boag, 2011). In this respect the structural theory has been criticized for reifying various activities into entities performing those same activities (Boesky, 1995) along with the further associated problem of describing the id, ego, and superego in anthropomorphic terms. Laplanche and Pontalis (1973), for instance, note that “[the structural theory] is shot through with anthropomorphism” (p. 452), which entails populating the mind with little people and then explaining the person with reference to persons – a tactic that simply multiplies the number of entities and explanations that need to be accounted for (Grossman and Simon, 1969; Wiedeman, 1972; Talvitie, 2012).

Furthermore, there are various problems with Freud’s aforementioned lines of demarcation between ego and id which extend the problems with the demarcations within his earlier topographic theory, *viz*. the supposed peculiar processes can be found across all of the systems (see Petocz, 1999). For instance, Brenner (1994) notes that “the ego is by no means as consistent, as integrated, as mature, and as immune from primary process functioning as the ego is supposed to be” (p. 477). On the other hand, the id cannot be ignorant of the external world since any id-impulse or urge can only be conceptualized as an impulse or urge to do, say, *X* or *Y* (where *X* and *Y* will be some real or imagined state of affairs; i.e., a content-less “urge” or “impulse” is incoherent). Moreover, any such urge or impulse is a propositional attitude (*S* desires that *X*) and so cannot be less organized or structured compared to any other mental act. In fact, Freud’s distinction between the ego and the id also generates problems for understanding the nature of the repressed since, as Beres (1962) notes, an “organized repressed content” must somehow then belong to the ego rather than the id:

If we assume that the fantasy which is unconscious retains its organization, to whatever degree, we must grant the continued activity of ego functions. “The ego is an organization,” Freud has said, “and the id is not.” An “id fantasy,” then, is by definition a contradiction in terms, and to speak of a fantasy being “repressed into the id” is, in my opinion, a complex of logical fallacies.

Beres, 1962, p. 324; cf. Slap and Saykin, 1984, p. 110

Furthermore, the id is clearly an agent like the ego (i.e., can initiate muscular activity) since id-impulses find their way into behavior as parapraxes and other compromise formations (e.g., Freud, 1935). The further supposed distinction contrasting the pleasure-seeking id with the self-preservative ego simply ignores the well-recognized fact that self-preservation and reality-orientation (which is only ever more or less) is itself a means of gratification and frustration avoidance (see Maze, 1983; Maze and Henry, 1996; Petocz, 1999; Newbery, 2011; Boag, 2012). As Maze and Henry (1996) note, the pleasure principle is the regulating guide or underlying motivational law of the mental apparatus for both the ego and the id. For that matter, the “reality principle,” too, is but a modified version of the pleasure principle since, as Freud notes, the ego “is able only to modify the pleasure principle but not to nullify it” (Freud, 1940, p. 198; cf. Freud, 1911b, 1925a). Accordingly, the reality principle is simply an elaboration of the pleasure principle and all mental activity is concerned with obtaining some sort of self-gratification even if appearing selfless or self-defeating.

While the problems of demarcation above have led some authors to reject the structural theory altogether (e.g., Brenner, 1994), none of this is to say that the ego and id cannot be differentiated by other means. Here at least one line of demarcation between ego and (repressed) id emerges in relation to the social context which is hostile toward certain means of gratification. Socially proscribed impulses become repressed and the resulting ego assumes a dominating position within the personality (Freud, 1900, pp. 594–595; Freud, 1905a, p. 85; Freud, 1907, p. 58). Nonetheless, given the problems of demarcation outlined above, the relationship between the ego and id requires clarification. Furthermore, the relationship between the ego and instinctual drives also requires further consideration.

## THE EGO AND THE DRIVES

The distinction between the ego and the id above led Freud to contrast the ego and the id in terms of motivational sources. More specifically, the id is motivated by the drives whereas the ego *manages* them: “For the ego, perception plays the part which in the id falls to instinct. The ego represents what may be called reason and common senses, in contrast to the id, which contains the passions” (Freud, 1923b, p. 25; cf. Freud, 1933, p. 76). Similarly, Freud (1923b) also writes that “[t]he ego develops from perceiving instincts to controlling them, obeying instincts to inhibiting them” (pp. 55–56). This disconnect between the ego and the drives leads to the distinction of the “irrational” id with the rational executive ego, analogous to a horse and rider:

The ego’s relation to the id might be compared with that of a rider to his horse. The horse supplies the locomotive energy, while the rider has the privilege of deciding on the goal and of guiding the powerful animal’s movements.

Freud, 1933, p. 77; cf. Freud, 1923b, p. 25

Maze (1987) notes that this distinction paved the way for the development of ego psychology whereby the ego is autonomous from the drives and consists of a set of functions including controlling motility, perceptual processes, synthetic functions, and an inhibitory capacity (Hartmann, 1950, 1958; Rapaport, 1951). While Hartmann (1950) initially believed that the “id and ego are originally one” developing “out of the matrix of animal instinct” (p. 79; cf. Freud, 1968, p. 59), he nevertheless conceptualized the developed ego as functionally distinct from the id. Later Hartmann (1958) discusses “inborn ego apparatuses” (p. 103) as well as “functions of the ego which cannot be derived from the instinctual drives” (p. 101), leading to the view of strict ego autonomy. The ego’s autonomy from the drives has subsequently become orthodox ego psychology [for a discussion of the history of ego psychology see Marcus (1999)]. Recently Lettieri (2005), for instance, refers to the “ego’s fundamentally autonomous, self-generating nature” (p. 377) and Gillett (1997) even distinguishes two autonomous egos: the “conscious ego” “similar to that of a central executive” and an “unconscious ego,” “also a central executive with functions limited to those required for the regulation of defense” (p. 482).

A general problem, however, with postulating an “irrational” id and “rational” ego is that this essentially paves the way for reinstating the Cartesian “rational faculty.” Here humans are divorced from the rest of the animal kingdom by virtue of “ego functions” which aim to manage, yet are independent of, the instinctual drives. Beres (1962), for instance, claims that “[h]uman psychic activity differs from that of animals, including, so far as we know, even the higher primates, by the mediation of ego functions between the instinctual drive stimulus, the need, and its gratification or inhibition” (p. 317). More recently Tauber (2010) has developed this implication to claim that the Freudian ego has free will and thus attributing to Freud a position he would find as antithetical to his favored deterministic position.

The more specific problem here with dissociating the ego from the drives is accounting for the ego’s motivation (Maze, 1983, 1987, 1993). Since the ego is said to arbitrate between different desires and demands (e.g., between the id, super-ego and external world – Freud, 1923b, p. 56), then some account of the ego’s motivational policy must be provided (why, for instance, does the ego choose to do *X* rather than *Y*?). Hartmann’s (1958) attempt to explain the ego’s motivation in terms of *adaptation* falls prey to an implicit moralism because adaptation is relative to different subjects’ points of view (see Maze, 1987). A similar criticism can be extended to Lettieri’s (2005) treatment of the ego as a “self-organizing adaptive process” (p. 376). Additionally, the ego becomes a truly free and autonomous agent, as reflected in the reference above to the “ego’s fundamentally autonomous, self-generating nature” (Lettieri, 2005, p. 377). Accordingly, and as Freud recognized, to avoid a disembodied rational agency, a motivational account ultimately based on some biological deterministic mechanism is required to explain both the direction and activity of any behavior.

## THE EGO AS A PORTION OF THE ID

While one line of Freud’s thinking paves the way to positing a drive-autonomous ego, Freud nevertheless provides a motivational basis for the ego when he writes that “[t]he ego is not sharply separated from the id; its lower portion merges into it” (1923b, p. 24; cf. 1933, p. 75). Similarly:

… this ego developed out of the id, it forms with it a single biological unit, it is only a specially modified peripheral portion of it, and it is subject to the influences and obeys the suggestions that arise from the id. For any vital purpose, a separation of the ego from the id would be a hopeless undertaking.

Freud, 1925a, p. 133

Freud’s reference here to a “single biological unit” is important here since it accounts for the ego’s motivation in terms of instinctual drives, in the same manner as the id and thus provides a possible explanatory motivational basis (Maze, 1983, 1987, 1993).

This position extends one line of thought in Freud’s thinking where he directly links the ego with the instinctual drives when he proposes the existence of “ego-instincts.” This position appears initially in one of Freud’s lesser known works (*The psycho-analytic view of the psychogenic disturbance of vision* – Freud, 1910) and appears to have enjoyed currency in Freud’s thinking for a relatively short period of time (1910–1915; Laplanche and Pontalis, 1973). The ego-instincts were broadly described as the “self-preservative” instincts (“hunger”) which could be contrasted with the libidinal instincts (“love”) and the account of ego instincts formalized this repressing source as a set of instincts responsible for conflict and subsequent repressions. Freud (1910) writes, for instance, that “instincts are not always compatible with one another; their interests often come into conflict. Opposition between ideas is only an expression of struggles between the various instincts” (pp. 213–214; cf. Freud, 1933, p. 57). Laplanche and Pontalis (1973) nevertheless note that although Freud had always postulated that it was the ego that initiated repression, until the formulation of the ego-instincts “the ego had until now been assigned no specific instinctual support” (p. 146). Freud’s account of the ego-instincts is thus theoretically welcome since it provides a biological foundation for the motivational systems involve in conflict (cf. Maze, 1983, 1987, 1993).

Laplanche and Pontalis (1973) note, however, an apparent inconsistency in Freud’s account of the ego-instincts. Freud (1910) describes the ego as a set of “ideas.” For instance, in describing the conflict between the repressed and the ego-instincts he writes that we “must assume that these ideas have come into opposition to other, more powerful ones, for which we use the collective concept of the ‘ego”’ (p. 213). This line of thinking, comparing the ego to a “dominant mass of ideas,” can be traced to Freud’s earliest psychodynamic writings (e.g., in the *Studies*, Freud refers to “the dominant mass of ideas constituting the ego” – Freud in Breuer and Freud, 1895, p. 116). Strictly speaking, however, ideas are policy-neutral and do not in themselves provide a basis for conflict. Laplanche and Pontalis further note here that treating the ego-instincts as a “group of ideas” is ambiguous in terms of whether the “ego” is either the *subject* or *object* of activity and cognition (i.e., the knower or something known). Laplanche and Pontalis question then whether the ego-instincts as “ideas” can serve as either the subject engaging in cognition or as motivational sources (“tendencies *emanating* from the organism” in their terms – p. 147, their italics), since, as “ideas”, the “ego” then is something *known* (the ego-instincts are “attached to the ego as if to their *object*”; Laplanche and Pontalis – p. 147, their italics). Laplanche and Pontalis believe that this apparent ambiguity was resolved with the introduction of the theory of narcissism. Insofar as the ego could be both subject and object, “[t]he ego-instincts emanate from the ego and relate to independent objects (such as food); yet the ego may become the object of the sexual instinct (ego-libido)” (p. 148; see also Freud, 1933, p. 58).

However, Freud’s position can be refined further. It is telling that Freud (1910) first mentions the ego in scare quotes (i.e., “ego”) in the context of a “collective concept” and a “compound” (p. 213. cf. p. 215). This is indicative of an attempt on Freud’s part not to reify the “ego” as a substantive agency or entity. After all, it is *multiple* instincts contributing to the collective, and it is this notion of multiplicity that helps conceptualize both splits and merging within the ego. Freud (1933), for instance, in discussing the ego as both subject and object writes that “the ego can be split; it splits itself during a number of its functions – temporarily at least. Its parts can come together again afterward” (p. 58). Freud (1910) further refers to “the collective concept of the “ego” – a compound which is made up variously at different times” (p. 213). That is, the ego is not an irreducible entity but rather composed of a dominating set of instinctual drives of which membership is fluid. For Freud (1910), what distinguishes the repressed instinctual drives from the instinctual drives composing the ego is that they remain isolated and incapable of synthesis into the collective forming the ego.

Freud’s ego-instincts account has, however, received little attention in psychoanalytic thinking and of those authors who do refer to ego-instincts, the tendency is to treat them, following following Hartmann (1958, p. 107), simply as synonymous with ego functions (e.g., Khantzian and Mack, 1983). One exception is Young-Bruehl and Bethelard (1999) who discuss both the history of the ego-instinct concept in Freud’s thinking and apply the concept to explaining object choice. Nonetheless, what is generally missing here is any substantive discussion of “instincts” generally to provide a foundation for understanding the ego-instincts. This is perhaps comprehensible given that Freud (1933) writes that “[i]nstincts are mythical entities magnificent in their indefiniteness” (p. 95), and that these instincts are “at once the most important and the most obscure element of psychological research” (Freud, 1920, p. 34; cf. Freud, 1905b, p. 168; Freud, 1925c, pp. 56–57*n*; Freud, 1930, p. 117). Furthermore, Freud’s editor Strachey’s choice of the term “instinct” as a translation for Freud’s “*Trieb*” is problematic since it connotes species-specific behavior patterns which is not in accord with Freud’s original usage (Laplanche and Pontalis, 1973; Ritvo and Solnit, 1995). “Drive” (or “instinctual drive”) has been discussed extensively elsewhere as a better translation (see Maze, 1983; Boag, 2012; Solms and Panksepp, 2012), and is the preferred term here.

## MAZE’S CLARIFICATION OF THE INSTINCTUAL DRIVE CONCEPT

A starting point for discussing drives is in the context of endogenous stimuli with respect to *source* (somatic process), *aim* (satisfaction), *object* (instrumental to aim), and *pressure* (motor) components (Freud, 1915a). Freud grounds drives somatically but relates them intimately to motivational states, cognitive activity and behavior. Drives engage in activity and for Freud the drives operate as endogenous stimuli, which, unlike external stimuli, persist until an activity or action is performed leading to satisfaction (i.e., the removal of the stimulus; Freud, 1895, pp. 296–297; Freud, 1905b, p. 168; Freud, 1915a, p. 118–119; Freud, 1925b, pp. 118–119; Freud, 1933, p. 96). While it is true that Freud chiefly speculated on the precise number and types of drives, this was not because drives are without real-world referents (as some claim – e.g., Fulgencio, 2005) but only because *psycho*analytic observations of behavior are limited with respect to what can be inferred about the source of any drive (Freud, 1915a). That is, the drive’s “source” means that drives must also be identified through investigating the internal workings of the body and not through psychological (psychoanalytic) enquiry alone.

Freud’s account of the instinctual drives has been variously criticized for invoking obsolete energic concepts (e.g., Holt, 1976; Rosenblatt and Thickstun, 1977) and not accommodating learnt experience (e.g., Westen, 1997). However, these criticisms rest upon outdated drive concepts, even if indicating a need for clarifying contemporary drive theory. This clarification has been achieved to some extent by the Australian psychoanalytic theorist Maze (1983, 1987, 1993). Maze conceptualizes the drives as neuro-physical “biological engines” that engage sensory and motor mechanisms and operate according to mechanistic, causal principles. As “engines,” the drives convert potential energy into output (behavior) and rather than initiating their own activity, are instead activated (switched “on”) by relevant bodily states (e.g., deprivation) and environmental conditions. Maze’s position is broadly consistent with current behavioral neuroscience accounts of drives (Sewards and Sewards, 2002, 2003; see Berridge, 2004 for a critical review; see also Bazan and Detandt, 2013 for a comparison of contemporary neuroscience and Freudian drive theory) whereby the complexity of human behavior emerges initially from innate consummatory activities (e.g., simple motor activities such as swallowing), which become elaborated through motor development and distorted due to factors such as conflict. For instance, socialization and culture shape how drives are expressed and any behavior may reflect a variety of motivational inputs such that several drives may contribute to any given activity. Behavior may also reflect conflict and repression, whereby compromised avenues of satisfaction are forced due to fear of punishment (see Maze, 1983; Petocz, 1999; Boag, 2012).

It should be noted here that Maze is not engaging in speculative theorizing and instead bases his proposal on the homeostatic drive accounts of his day, a position still found in current behavioral neuroscience, even if possibly not covering the whole story (for instance, allodynamic processes may be needed to supplement homeostasis – Schulkin, 2003; McEwen and Wingfield, 2010; see also Berridge, 2004). Furthermore, current behavioral neuroscience provides in principle support for Freud’s (1910) libidinal and self-preservative drive distinction, with drives of sexuality, hunger, thirst, pain reduction, sleep, fear, power-dominance, and nurturance identified (Wagner, 1999; Sewards and Sewards, 2002, 2003). However, the aim here is not to provide an exhaustive list of drives (and any satisfactory list will necessarily require careful scrutiny) but simply to show that an account of drives conforming to Freud’s initial postulation is not entirely without contemporary justification.

Since Freud’s time there are also broader questions concerning whether affects should be considered the primary motivational instigators rather than the drives (see Westen, 1997; Kernberg, 2009) and while there is not scope here to develop this issue, a motivational account of affects does not necessarily contradict the Freudian drive position since drives and affects appear to be intimately connected (Boag, 2008, 2012, Chap. 8; McIlwain, 2007). On the other hand, whether aggression is a primary drive or a reactive response of the drives to frustration remains an open-question and is also subject to ongoing debate (see Kernberg, 2009). Although some authors consider Freud’s (1920, 1923a) concept of a death instinct clinically useful (e.g., Segal, 1993), or useful in conjunction with a notion of a primary aggressive drive (e.g., Kernberg, 2009), a problem with Freud’s life and death drives is that these drives are defined by aim rather than source, and the question remains, as Kernberg observes, as to whether any observed aggression is a primary drive or a secondary response to frustration. Without bio-neurological evidence of a primary death/aggressive drive (i.e., a source) then one and the same aggressive expression can be “explained” in terms of it being either an expression of a primary aggression drive or reactive aggression (in other words, we are left guessing whether aggression is a primary drive requiring satisfaction or not). Nevertheless, it is still possible to agree with Kernberg (2009) that aggression is a “major motivational system” (p. 1018) since aggression appears to be part of our make-up, but whether we have primary aggressive aims that require satisfaction (rather than simply reactive aggression) remains to be seen.

## DRIVES AND COGNITION

Maze (1983) further aims to clarify the relation between drives and cognition. Any act of cognition entails something standing as a *knower*, and while one commonly refers here to the “person” or organism, an innovation in Maze’s thinking (again following hints from Freud) is that it is the drives themselves that engage in cognition. In other words, the organism’s motivational systems are what engage cognitive/perceptual processes. This is, at times, indicated by Freud when he treats the drives as the “psychical representative of organic forces” (i.e., wishes or desires; Freud, 1911a, p. 74; cf. Freud, 1915a, p. 122; Freud, 1905b, p. 168), and elsewhere when Freud writes that an “instinct can never become an object of consciousness – only the idea that represents the instincts can” (Freud, 1915c, p. 177). This could be taken to mean that the drives are defined somatically but simultaneously engage in cognition in their quest for gratification and avoidance of frustration (e.g., a hunger drive desires to eat and is interested in sources of food – Passmore, 1935). More recently, Panksepp (1998) to some extent suggests a similar position whereby he appears to imply that drives engage both cognition and behavior via the SEEKING system: “The SEEKING system, under the guidance of various regulatory imbalances, external incentive cues and past learning, helps take thirsty animals to water, cold animals to warmth, hungry animals to food, and sexually aroused animals toward opportunities for orgasmic gratification” (p. 167; cf. Panksepp and Moskal, 2008; see also Bazan and Detandt, 2013, pp. 6–7; see also Boag, 2012, pp. 114–115). Accordingly, the drives can be considered as psychobiological systems rather than “blind bodily forces” as some propose (e.g., Slavin and Grief, 1995, p. 166). It is the drives that desire, believe, and phantasise, and such desires, beliefs, and phantasies are not automatically conscious and may be prevented from being reflected upon via repression (Freud, 1915b; for further discussion see Boag, 2010, 2012, Chaps. 5 and 6).

Subsequently, Maze (1983, 1987) develops the position that the smallest units comprising the “knowers” within the personality are the psychobiological drive structures: “Each instinctual drive accumulates information and misinformation about the location and means of acquisition of the objects necessary for its specific actions to be performed” (Maze, 1983, p. 162). While not a common standpoint, this view has nevertheless been taken up by others to account for the dynamics of mental life (e.g., Petocz, 1999; Boag, 2005, 2012; Newbery, 2011) and although we can generally refer to the “organism” as that which knows, the fact of psychological conflict – a foundation of psychoanalytic theory – forces upon us the view of there being multiple systems, each motivated and cognising, coming into conflict (a position not dissimilar to Plato’s observation that human life reflects “struggles of factions in a State” – Plato, 1928; 440b, p. 170). As Petocz (1999) writes, “in order to accommodate the facts of mental conflict, of a conflict of interests within a single mind, there must be a plurality of drives – at least two” (p. 221; cf. Maze, 1983; Boag, 2005, 2007, 2012; McIlwain, 2007). That is, it is literally possible to be in “two minds” about something since, unlike the indivisible and immaterial Cartesian ego, any experience of “self” belies what is in fact a multiplicity of knowing systems.

Maze is thus proposing a strongly partitive view of personality: each individual is made up of a small community of these drives, “each of which is a knower and a doer” (Maze, 1987, p. 197). However, Maze is not suggesting that these drives are anthropomorphic homunculi (“little persons”). Instead, these knowers are biomechanical systems utilizing cognition:

…unlike the whole person each has, in effect, only one motive, never restrains itself from seeking satisfaction, knows only a portion of the aggregate body of information, and suffers no internal conflict. An instinctual drive can no more restrain itself from working than any motor can, once the switches are thrown. If its operation is to be arrested, then that must be through some influence external to itself – in the case of repression, from other instinctual drives

Maze, 1987, p. 197

Thus, rather than a “person” (or persons) acting rationally and deciding upon courses of action, each drive is simply a mechanistic system that once activated motivates the organism’s cognitive activity and behavior. The restraining and inhibiting factors are consequent on conflict and repression (Maze, 1983; Boag, 2012) and the behavior of the “person” results from both facilitating and inhibiting influences emerging from the interaction of these drives.

A potential shortcoming with Maze’s account has, however, been identified by Newbery (2011) with respect to accounting for the ongoing activity of the organism after apparent satiation. In Maze’s account, once drive-satiation occurs then the consequent cessation of the drive-excitation pattern would terminate stimulation of motor activity (Maze, 1983, p. 151). Accordingly, should all the primary drives be satisfied then the organism would cease to engage in motivated behavior. Addressing this issue, Newbery proposes a conceptual distinction between drives being *inoperative* – ceasing activity altogether – and drives being *satisfied*, whereby the drives nonetheless engage in cognitive activity even when satisfied (Newbery, 2011, p. 857). Here Newbery postulates that a satisfied drive may nevertheless remain sensitive to environmental sources of gratification and frustration. A hunger-satiated person may remain alert to hunger-gratifying information when, for instance, environmental information affords the possibility of predicting food shortages.

## EGO-DRIVES AND ID-DRIVES

Following Freud’s (1910) ego-instinct account, Maze (1983, 1987) proposes that both the ego and the id are constituted by the drives. Furthermore, both the ego and id engage in acts of knowing. Consistent with Freud’s position, the primary factor determining which sub-set of drives form the ego-drives and which form the id are the social forces instigating repression. In Freud’s theory it is primarily the social environment during infancy which provides the context for repression (Freud, 1914, 1915b). Wishes and desires that are proscribed by the social environment are associated with loss of love and danger, leading to anxiety and primary repression. Later, such external prohibitions become internalized as the superego, reinforcing the initial repression with secondary repression (Freud, 1926, p. 128; Freud, 1930, p. 124). Here Maze (1987) writes that the drive expressions (i.e., desires and behaviors) that provoke anxiety due to associations with “loss of love” form the repressed id while the ego emerges as a dominating sub-set of the drives:

In general, all those instinctual drives whose gratification is dependent on the parent’s good will and which is employed as reward by them are mobilized in opposition to the forbidden instinctual impulses. Thus, one subset of the instinctual drives becomes organized in competition with the remainder, and treats the blocking off of the remainder as an essential part of securing its own gratification

Maze, 1983, p. 171

While Freud (1910) viewed the self-preservative drives as those constituting the ego-drives and the libidinal drives as those constituting the repressed, Freud further notes that although the sexual drives are typically repressed, this is essentially an empirical finding rather than an *a priori* assertion (for the role of sexuality in personality and repression see also Boag, 2012, pp. 118–119).

Theoretically there is no objection to supposing that any sort of instinctual demand might occasion the same repressions and their consequences: but our observation shows us invariably, so far as we can judge, that the excitations that play the pathogenic part arose from the component instincts of sexual life

Freud, 1940, p. 186

Viewed in this way there are no *a priori* “id” drives and Bleichmar (2004) rightly notes that it is not inconceivable that sexuality could repress the aim of self-preservation (p. 1387). What becomes repressed is determined by prevailing social factors and inter-drive competition and it is moral disapproval that provides the context for the development of id and ego:

The actual principle of division, between the instinctual drives that are to constitute the ego and those which are to be repressed and constitute the id, would be that the former were those whose expression was not subject to *moral disapproval*, that is, those which were socially regarded as legitimate constituents of a respectable person, whereas the latter were morally condemned as impulses that no worthwhile person would have

Maze, 1983, p. 172, his italics

Given then that the ego is not divorced from the drives, the id is better conceptualized solely in reference to what is repressed. Furthermore, the ego and id are not then fixed and unchanging entities. For both Freud (1910) and Maze (1983), the “ego” is not a substantive, indivisible entity but rather a fluid collective of dominating drives that have not submitted to repression. Accordingly, there is no single executive entity called the ego acting as the agency of repression. Instead, the protagonists behind repression are the ego-drives, guided by beliefs of frustration and gratification and preventing id-drives from forms of expression. Should the social context change (and with it the conditions of moral reproach), then the inclusion within ego and id would also be subject to change (cf. Maze, 1983).

## EGO, IDENTIFICATION, AND IDENTITY

Given that the ego is not an indissoluble entity but instead a collection of dominating drives, some account is required for explaining how a sense of identity forms normatively to form a singular sense of “self.” Gardner (1993), in fact, objects to the possibility of multiple knowers given that phenomenologically we only have a single subjective frame of reference and can only ever experience ourselves as a single person (p. 83). While this is disputable (e.g., there are a variety of dissociative phenomena that suggest otherwise – see Kluft, 2000), the “single frame of reference” criticism instead provides the answer to why we do not recognize multiplicity. Given that the drives are *brain structures* (connected nevertheless intimately with various physiological organs, perceptual systems, etc.), there is ordinarily no possibility of direct awareness of the drives themselves. However, the expressions of the drives are knowable, and such expressions are realized through the body and its various activities (including cognition). As Freud (1923b) notes, the known ego “is first and foremost a body-ego” (p. 27) and “the ego is ultimately derived from bodily sensations” (p. 26*n*). Here the distinction between the ego-drives and the “known ego” or ego-self is important. Solms and Panksepp (2012) contribute here the important point that *awareness of the body* is the same as awareness of objects in the world generally. The body in this sense is something known (including fantasized about), and so a sense of unity rather than division is what is apparent. Freud (1910) additionally notes that both the ego-drives and sexual drives have an anaclitic relationship: “The sexual and ego-instincts alike have in general the same organs and systems of organs at their disposal” (Freud, 1910, pp. 215–216; cf. Freud, 1912, pp. 180–181). Accordingly, what the drives know in relation to themselves is the “person’s” desires, beliefs, and actions (a shared pool of beliefs): The apparent unity of the ego follows from a drive neither knowing itself directly (nor the other drives) and instead only knowing the organism generally and its activities via a singular perceptual apparatus of which each drive partakes in. As I elsewhere note (Boag, 2005), “[t]he resulting belief of a unified self is as *prima facie* plausible as the belief that the sun revolves around the earth” (p. 753). Our social interactions further reinforce developing a coherent sense of self since we appear as a single organism to others and are treated as such accordingly.

However, one’s sense of self of course is not simply restricted to the actual organism and can extend further than the body. Here the role of *identification* in the formation of the ego-self is paramount. On the account of ego-drives here, it is the drives themselves that are the subjects identifying with different states of affairs. While the drives typically identify with the body and psychic apparatus, such identification can conceivably extend to family and possessions and beyond. James (1890/1950) was one of the first to recognize this in modern psychology where, in his discussion of the “self”, he writes:

*In its widest possible sense* ….* a man’s Self is the sum total of all that he* CAN* call his*, not only his body and his psychic powers, but his clothes, and his house, his wife and children, his ancestors and friends, his reputation and works, his lands and horses, and yacht and bank-account. All these things give him the same emotions. If they wax and prosper, he feels triumphant; if they dwindle and die away, he feels cast down… (p. 291, italics/capitals in original).

“Personal identity,” then, can be seen as a collective viewpoint whereby a subset of the drives identifies with (most commonly but not necessarily) the body and its parts, other people to various degrees, values, society, culture, etc. Nevertheless, the known ego-self is essentially a fantasy – a false belief – based on appearances (cf. Grossman, 1982; Solms and Panksepp, 2012). This ego-self, while appearing as an agent, is simply a puppet driven by the ego-drives.

## THE SUPEREGO AND THE POSSIBILITY OF MULTIPLE EGOS

Given that the ego is conceptualized above in terms of the drives and their identifications the question arises then concerning the nature of the superego and other possible dynamic structures. On Maze’s (1983, 1987, 1993) view the superego is not a dynamic structure (or agent, or independent set of drives) and instead conceptualized in terms of the moral *beliefs* guiding the ego in terms of possible sources or gratification and punishment (see also Boag, 2006, 2012), a position reflecting Brenner’s (1994) insistence that there is no special agency of the superego. On this view, the superego is not a motivated, cognising agency but simply internalized obstructions in the form of beliefs, even if represented by personal forms based on identification with actual people.

However, one limitation of the “superego as beliefs” account here is that the theory does not appear to account for the observed clinical phenomena. More specifically, treating the superego merely as a set of moral beliefs contradicts Freud’s own descriptions (and others after him) whereby the superego itself appears to be both motivated and active:

In the course of an individual’s development a portion of the inhibiting forces in the external world are internalized and an agency is constructed in the ego which confronts the rest of the ego in an observing, criticizing and prohibiting sense. We call this new agency the *super-ego*. Thenceforward the ego, before putting to work the instinctual satisfaction demanded by the id, has to take into account not merely the dangers of the external world but also the objections of the super-ego, and it will have all the more grounds for abstaining from satisfying the instinct.

Freud, 1939, pp. 116–117, his italics; cf. Freud, 1914, p. 95; 1916–1917, pp. 428–429; Freud, 1933, p. 60

If Freud’s account of the superego as a dynamic structure is to be taken as an accurate reflection of the psychic situation, then some dynamic account of the superego’s motivational sources is required. Rather, then, than treating the superego simply in terms of beliefs (or even phantasy), the account to be developed here proposes that the superego is dynamic (motivated) and is based on a differentiation of the drives organized around (i.e., identified with) a parental imago.

A drive position can be reconciled with Freud’s position on the superego above through proposing that the superego entails a subset of the ego-drives reflecting *intra-ego* conflict. Although this is not Maze’s position, if the ego is a sub-set of the drives as Maze proposes, then there is no logical objection to there being further divisions. Proposed here, then, is that a subset of the ego-drives, the *superego-drives*, identifies with the external inhibiting sources based on a self-preservative motive and becomes (in certain situations) turned against the remainder of the ego-drives (and the id). Accordingly, the superego is a motivated organization, in the same way as the ego, and the moral character of the superego follows from the characteristic finding that this superego subset of the drives identifies with the moral authority of the caregivers (Freud, 1923b, p. 36; see also Pulcu, 2014, for a recent discussion of superego formation within an evolutionary context).

If this account of superego-drives is granted, then by logical implication other personality divisions are possible. That is, if various drives can join forces but can also be in conflict, then there is the possibility of divisions and further sub-divisions of dynamic structures based on various identifications giving rise to a multiplicity of “personalities.” Freud (1923b), in fact, directly addresses this when discussing the superego and the formation of the ego’s object-identifications. There Freud observes that the ego’s multiple object-identifications may become “too numerous, unduly powerful and incompatible with one another” and that “perhaps the secret of the cases of what is described as “multiple personality” is that the different identifications seize hold of consciousness in turn,” a position he describes as not necessarily pathological (pp. 30–31)^1^. Indeed, although the id, ego, and superego are commonly observed personality structures, Freud proposes that variations may nevertheless be found:

In thinking of this division of the personality into an ego, super-ego and an id, you will not, of course, have pictured sharp frontiers like the artificial ones drawn in political geography… After making the separation we must allow what we have separated to merge together once more… *It is highly probable that the development of these divisions is subject to great variations in different individuals*…

Freud, 1933, p. 79, emphasis added

Accordingly, the triadic structural theory is simply typical rather than inevitable: various other personality divisions might arise in the same manner as the account of id, ego and superego here. Consequently, given that the superego can represent a sub-set of the ego-drives which act as an apparent agent, there is no logical problem with the conclusion then that multiple “egos” could potentially form which, for all intents and purposes, reflect “multiple personalities” [a possibility also briefly touched upon by Freud (1914) in *On narcissism*]. Here various sub-sets of drives could constitute various “egos,” each developing an independent and separate sense of identity or self-hood via various object-identifications.

What the present account contributes then is a dynamic framework for understanding a variety of normal and clinical psychological phenomena related to “multiple personalities” including Dissociative Identity Disorder (DID). DID manifests as disruptions in identity characterized by two or more “distinct personality states” (possibly experienced as “possession”) and is associated with recurring gaps in remembering of events including trauma (APA, 2013, p. 292). While DID finds inclusion in the DSM-5 there is nevertheless persistent controversy as to whether DID is in fact an authentic disorder (see, for instance, Piper and Merskey, 2004a, b; Boysen and VanBergen, 2013, 2014; for recent examples of the controversy surrounding fantasy and trauma models, see Dalenberg et al., 2012, 2014; Lynn et al., 2014). One major theoretical problem within this controversy concerns how best to conceptualize personality within DID, an issue that also impacts upon clinicians working with DID with respect to conceptualizing the personalities (“alters”) and then engaging with them in therapy: “Among the major issues that arise in the treatment of DID are the relationship of the alters to the personality that the therapist may experience as his or her patient, and the relationship of the therapist to the alters” (Kluft, 2000, p. 266). As a consequence of this uncertainty, Kluft writes that many psychoanalytic clinicians (even if accepting that some version of alters exist), “are reluctant to address them [the alters] directly or to request or facilitate their participation in the process” (p. 270). On the theoretical position developed above, there is theoretical justification for treating alters in the same manner as one would treat the “ego”: both the ego and alters can be thought of as drive-object-identifications and neither are indivisible nor fixed. Accordingly, and given the centrality of the therapeutic alliance (Meissner, 2007), there is justification for Kluft’s (2000) proposal of forming therapeutic alliances with the alters (i.e., the clinician addresses the alters as one would the “ego” and engages them in the therapeutic process). Clinical issues aside, a further implication from the account proposed here is that (at least in principle) there may be further means of contributing to neuroscientific studies addressing the authenticity of DID (e.g., Reinders et al., 2012; Schlumpf et al., 2013). Given that drives essentially entail neural sources (see Bazan and Detandt, 2013) then their contribution to DID should be assessable, in the same manner as the motivational contributions to dreaming are presently being assessed (e.g., Colace, 2014; Colace and Boag, in press).

## EGO-DRIVES, OBJECT-RELATIONS, AND THE DYNAMICS OF INTERNAL OBJECTS

Placing primary emphasis upon the drives raises questions concerning how this account fits with an object-relations approach which views motivation primarily in terms of developing relationships with others (e.g., Fairbairn, 1952; Guntrip, 1968; Greenberg and Mitchell, 1983). Maze (1993) rightly argues that drive accounts and object-relations theory are not, as commonly supposed, antithetical since the drives can be seen as the subjects involved in the motivated, emotional relationships with objects. Furthermore, a drive account provides a biologically grounded explanatory framework for understanding why a person is motivated to relate to certain objects and not others, as well as helping understand the dynamics underlying frustration and gratification within object-relationships. In addition to this, Young-Bruehl and Bethelard (1999) have gone some way to applying an ego-drive account for understanding the relationship between the developing infant and actual caregivers, and thus demonstrating that an ego-drive account can accommodate an object relations perspective. What is less certain, however, is the relationship between drives, ego structures, and *internal objects*.

While object-relational approaches are diverse, one influential approach is that of Fairbairn (1952) which has been developed by Ogden (1993, 2002, 2010) and also discussed in contemporary philosophy of psychoanalysis (e.g., Pataki, 2014). As Ogden (1993) notes, while object-relations are commonly thought of in terms of interpersonal relations, it “is in fact fundamentally a theory of unconscious [intrapersonal] object relations in dynamic interplay with current interpersonal experience” (p. 131, my insertion; see Ogden, 1993, p. 131*n*). These intrapersonal relationships result from a fragmenting of the ego leading to a multiplicity of ego-structures and their internal objects. For Fairbairn (1944/1952) an initial unitary Central ego becomes split into separate dynamic egos (Central ego, anti-libidinal (internal saboteur) and libidinal egos) each existing as dynamic (motivated) “endopsychic structures.” This differentiation of the ego is a consequence of frustration and repression, leading to split-off aspects of the Central ego attached to their frustrating internal objects (see also Ogden, 2010).

For Maze (1993), however, internal objects are simply imagined entities that have no actual autonomy and exist simply as objects of a person’s desires and beliefs. He writes that despite views to the contrary “[i]nternal objects do not really initiate their own behavior” (Maze, 1993, p. 464), and while it may appear that internal objects act autonomously, this, according to Maze, is more apparent than real:

… while this way of describing them may convey the patient’s phenomenological experience very powerfully, it seems to me to obscure the fact that one is really dealing with the person’s thoughts, wishes and fears, or more precisely, with the beliefs, wishes and fears of various parts of the person’s mind

Maze, 1993, p. 464

Nevertheless, the observation of apparent autonomous centers within the psyche is not uncommon and the notion of multiple autonomous inner personalities is found in a variety of theories. For instance, Symington (1993) writes that “[w]e are all made up of parts, each part capable of functioning as a separate little person” (p. 23), and, within the context of object-relations he writes: “when we talk about an internalized mother, father, brother, sister, or whatever, these are internalized objects, and these objects acts. They act within the personality. At certain points they may even take over the personality” (p. 20). The question then is how we can account for these apparent autonomous centers that addresses both the phenomenology and provides a satisfactory explanatory framework.

## EGO-STRUCTURES AND INTERNAL OBJECTS

The relation between ego-structures and internal objects is not always clear in Fairbairn’s theory and while Fairbairn generally treats ego-structures as dynamic he does not consistently do so with internalized objects (Maze, 1993; Ogden, 1993). Ogden (1993) writes that “Fairbairn only hesitatingly accepted the idea that internal objects are dynamic structures and was not able to delineate the relationship between the concept of internal objects and the concept of the ego” (p. 156). For example, whereas Freud treats the superego as a dynamic structure, Fairbairn in places explicitly does not [e.g., Fairbairn, 1944/1952, pp. 131–132; see also Maze (1993, p. 463)]. Nevertheless, Fairbairn can also be found to grant autonomy to both various ego structures *and* internal objects, which he sees as an extension of Freud’s position that the superego is an autonomous structure:

… the time is now ripe for us to replace the concept of “phantasy” by a concept of “inner reality” peopled by the ego and its internal objects. These internal objects should be regarded as having an organized structure, an identity of their own, an endopsychic existence and an activity [a capacity for thinking and feeling] as real within the inner world as those of any objects in the outer world. To attribute such features to internal objects may at first seem startling to some; but, after all, they are only features which Freud has already attributed to the superego… What has now emerged is simply that the superego is not the only internal object

Fairbairn, 1943, in Ogden, 2011, p. 938, Ogden’s insertion

Ogden (2011) takes this to mean that internal objects are identical with ego-structures, writing that for Fairbairn, internal objects are not merely phantasy or simply ideational content: “Internal objects are not ideas – they are split-off parts of the ego with which the internal world is “peopled”” (p. 938). Furthermore, as dynamic agents internal objects are the subjects that do the phantasying: “*phantasying is the product of internal objects (*i.e.,* internal objects are the thinkers doing the unconscious thinking)*” (Ogden, 2011, p. 139, his italics). On the other hand, if, as Maze (1993) believes, internal objects are primarily beliefs, phantasies, and desires, then such internal objects cannot exists as autonomous structures simply because beliefs, phantasies, and desires are activities and so cannot be structures engaging in those same activities (see also Boag, 2005).

Ogden’s intention here is to clarify the relation between ego-structures and internal objects and address a short-coming in Fairbairn’s approach, viz. Fairbairn “did not explain how an internal object (presumably originally thought) achieves its dynamism” (Ogden, 1993, p. 148). Nevertheless, identifying ego-structures with the internal objects does not mean that ego-structures and internal objects cannot be differentiated, even if “[t]his identification with the object is so thorough that one’s original sense of self is almost entirely lost” (Ogden, 1993, p. 132). To clarify this, Pataki (2014) proposes that the ego-structures are “attached” to their respective objects, and that these internal objects are dynamic by virtue of their relation to these ego structures. Fairbairn (1951/1952) similarly writes:

Although I have spoken of internalized objects as structures, I have treated them simply as objects of dynamic ego-structures, and not as themselves dynamic. … In the interests of consistency, however, I must draw the logical conclusion of my theory and acknowledge that, since internal objects are endopsychic structures, they must be themselves in some measure dynamic; and it should be added that *they must derive their dynamic quality from their cathexis by ego-structures*

Fairbairn, 1951/1952, p. 177; my emphasis

Thus, Fairbairn could be taken to be proposing an ego-structure/phantasy-object relationship whereby the internal object is itself phantasy but nevertheless dynamic insofar as the respective ego-structure identifies with the phantasy object (cf. Pataki, 2014). Pataki (2014) describes this identification between ego-structures with internalized figures as *personation* which I take to mean that the ego-structures take on (personate) actual or phantasized individuals.

Pataki (2014) observes that the possibility of multiple ego-structures/internal objects implicated in the account above raises complex questions with respect to understanding agency, personhood and identity. Furthermore, whether we should commit to a theory of multiple “persons” inhabiting the psyche requires careful scrutiny. After all, Maze (1993) proposes a simpler explanation whereby internal objects are nothing more than phantasy reflecting fears and desires, rather than constituting distinct centers of agency. Accordingly, if Fairbairn and Ogden are to be believed then a theoretical basis for postulating ego-structures and their internal objects as dynamic structures (capable of acting and knowing) is required. Here Ogden (1993) claims, though, that his account of internal objects “goes no further in the direction of demonology than did Freud in describing the formation of the superego” (p. 150). However, this is not necessarily a virtue. As noted earlier, Freud’s theory of the id, ego and superego has been heavily criticized for both reification and anthropomorphism (e.g., Grossman and Simon, 1969; Laplanche and Pontalis, 1973; Boesky, 1995; Talvitie, 2012). As with Freud, both Fairbairn and Ogden are open to the criticism that their account of the ego-structures simply multiplies instances of reification since both Fairbairn and Ogden tell us what the various ego-structures *do* but not what they actually *are* (and so leave the ego-structures uncharacterized).

The ego-drive account developed earlier provides a theoretical rationale for understanding ego-structures and their internal objects as dynamic organizations. The ego-structures are characterized in terms of subsets of the (biologically grounded) drives which, in turn, provides a basis for making sense of both personality differentiation and the motivational bases of ego-structures and their internal objects. As argued earlier, there is no logical difficulty in proposing various drive-combinations organized around various apparent centers of agency (ego-structures, etc.). Internal objects reflect combinations of drives forming dynamic structures identifying with an imago and acting as an apparent agent. This is “apparent” simply because while the drives constitute the knowing motivational systems, the drives underlying the various ego-structures mistakenly see themselves as “persons” acting as a singular agent. Consequently, given that Fairbairn *does* aim to provide an explanatory system for the dynamics of endopsychic structures (e.g., Fairbairn, 1944/1952, pp. 128ff), Fairbairn’s account gains everything from admitting a drive basis to account for the motivation of ego-structures and loses nothing with respect to providing his distinctive object-relations approach.

## DREAMS AND INTERNAL OBJECTS: EVIDENCE FROM CASE STUDIES

The dynamics of dream figures provides an indication of apparent structures or agents with their own dynamics, a finding which, *prima facie*, contradicts Maze’s (1993) proposal that internal objects are simply objects of imagination and not separate organizations. In discussing the dreams of a patient with a physical genital abnormality, Fairbairn (1931/1952) describes how two dream figures appeared to correspond to the id and superego (“the mischievous boy” and the “critic,” respectively – p. 217). These dream figures were also active, to the point where “invasion of waking life by [these] personifications did actually occur” (p. 219). For instance, at the onset of analysis “‘the mischievous boy’ took almost complete possession of her conscious life” (p. 219). Nevertheless, Fairbairn writes that “[i]t must be recorded, however, that the dream-figures so far mentioned by no means exhaust the personifications appearing in this patient’s life” (p. 217), and during analysis other figures (e.g., a “little girl” and “the martyr”) also emerged. Consequently, Fairbairn writes that the prevalence and multiplicity of such dream figures raises questions concerning whether Freud’s tripartite division has led us to consider the id, ego and superego “too much in the light of entities” (p. 218). This is an important observation because it indicates how personality structures are commonly treated as stable and fixed entities when, instead, the findings suggest that the psyche and its ego-structures and internal objects are fluid and maleable. What is required then is a theoretical approach that can account for the motivation of dynamic structures but is also flexible enough to accommodate both ongoing personality division and combination resulting in various organizations within the psyche. An ego-drive approach provides a basis for understanding both these splits within personality as well as how drive combinations could give rise to a variety of personifications. On the account developed here, neither the ego, id, and superego, nor ego-structures and internal objects are immutable. Instead, and following a line of Freud’s own thinking, “the ego can be split; it splits itself during a number of its functions – temporarily at least. Its parts can come together again afterward” (Freud, 1933, p. 58). The relative fluidity of drive-subsets organizing themselves around an imago (a fluidity which seems to be further facilitated in dreaming) is central here to understanding the complexity of situations faced by the clinician. Furthermore, viewing internal objects as based on drives indicates why the figures in dreams express their own frustration and gratification (cf. Maze, 1993) and how these structures, too, can be instigators of repression in a psyche where the various ego-structures facilitate or attempt to cancel one another out. Consistent with Fairbairn’s theory (e.g., Fairbairn, 1943/1952, 1944/1952) repression involves the ego rejecting aspects of itself and provides the grounds for splitting the personality into various dynamic structures within the psyche (cf. Ogden, 1993, 2010).

## CONCLUSION

This paper contributes to a metapsychological understanding of the id, ego, and superego through developing Maze’s ego-drive account based on Freud’s (1910) ego-instincts. On this view the ego emerges as a sub-set of drives which have achieved control over another sub-set of drives that form the (repressed) id. The drives that constitute the ego develop a sense of identity (self-hood) whereby the ego-drives identify with various states of affairs in unison, which for the most part includes a view of a singular organism (“person”). A theoretical innovation here is that there is no logical objection to there being multiple egos within any individual as found in Fairbairn’s object-relations account. Furthermore, extending this ego-drive account to object relations theory provides both a synthesis of otherwise distinct positions and an explanatory basis for ego-differentiation and the dynamics of ego-structures and internal objects. While the fore-going discussion is primarily theoretical, both conceptual and theoretical research is essential for developing psychoanalysis generally, and particularly with respect to clarifying and refining our understanding of psychoanalytic theory and systems (see also Dreher, 2005; Leuzinger-Bohleber and Fischmann, 2006). This ego-drive account goes some way to providing a theoretical framework that bridges the “wide gulf between the actual theories that clinicians apply in their work and the psychoanalysis that is learned in universities” (Symington, 1993, p. 4) through providing an account of persons, internal or otherwise, that is consistent with a coherent theory of motivation as embodied in Freud’s (1915a) metapsychology of the instinctual drives.

## Conflict of Interest Statement

The author declares that the research was conducted in the absence of any commercial or financial relationships that could be construed as a potential conflict of interest.

## Footnotes

1 I would like to thank a Reviewer for drawing my attention to this reference.

## References

[B1] Bazan A. Detandt S. (2013). On the physiology of jouissance: interpreting the mesolimbic dopaminergic reward functions from a psychoanalytic perspective. *Front. Hum. Neurosci.* 7:709 10.3389/fnhum.2013.00709 PMC3818686 24223543

[B2] Beres D. (1962). The unconscious fantasy. *Psychoanal. Q.* 31 309–328 14009075

[B3] Berridge K. C. (2004). Motivation concepts in behavioral neuroscience. *Physiol. Behav.* 81 179–204 10.1016/j.physbeh.2004.02.004 15159167

[B4] Bleichmar H. (2004). Making conscious the unconscious in order to modify unconscious processing: some mechanisms of therapeutic change. *Int. J. Psychoanal.* 85 1379–1400 10.1516/PDAK-M065-JEUJ-J7EQ 15801514

[B5] Boag S. (2005). Addressing mental plurality: justification, objections & logical requirements of strongly partitive accounts of mind. *Theory Psychol.* 15 747–767 10.1177/0959354305059022

[B6] Boag S. (2006). Freudian repression, the common view, and pathological science. *Rev. Gen. Psychol.* 10 74–86 10.1037/1089-2680.10.1.74

[B7] Boag S. (2007). Realism, self-deception and the logical paradox of repression. *Theory Psychol.* 17 421–447 10.1177/0959354307077290

[B8] Boag S. (2008). ‘Mind as feeling’ or affective relations? A contribution to the School of Andersonian Realism. *Theory Psychol.* 18 505–525 10.1177/0959354308091841

[B9] Boag S. (2010). Repression, suppression, and conscious awareness. *Psychoanal. Psychol.* 27 164–181 10.1037/a0019416

[B10] Boag S. (2011). Explanation in personality research: ‘verbal magic’ and the five-factor model. *Philos. Psychol.* 24 223–243 10.1080/09515089.2010.548319 PMC4241598 25431525

[B11] Boag S. (2012). *Freudian Repression, the Unconscious, and the Dynamics of Inhibition*. London: Karnac

[B12] Boesky D. (1995). “Structural theory,” in *Psycho-analysis: The Major Concepts* edsMoore B. E. Fine B. D. (New Haven: Yale University Press) 494–507

[B13] Boysen G. A. VanBergen A. (2013). A review of published research on adult dissociative identity disorder: 2000–2010. *J. Nerv. Ment. Dis*. 201 5–11 10.1097/NMD.0b013e31827aaf81 23274288

[B14] Boysen G. A. VanBergen A. (2014). Simulation of multiple personalities: a review of research comparing diagnosed and simulated dissociative identity disorder. *Clin. Psychol. Rev.* 34 14–28 10.1016/j.cpr.2013.10.008 24291657

[B15] Brakel L. A. W. (2009). *Philosophy, Psychoanalysis, and the A-Rational Mind*. Oxford: Oxford University Press 10.1093/med/9780199551255.001.0001

[B16] Brakel L. A. (2013). *The Ontology of Psychology: Questioning Foundations in the Philosophy of Mind*. London: Routledge

[B17] Brenner C. (1994). The mind as conflict and compromise formation. *J. Clin. Psychoanal.* 3 473–488

[B18] Breuer J. Freud S. (1895). *Studies in Hysteria (1893–1895)* Standard Edn Vol. II. London: Hogarth

[B19] Colace C. (2014). *Drug Dreams: Clinical and Research Implications of Dreams about Drugs in Drug-addicted Patients*. London: Karnac

[B20] Colace C., Boag S. Persisting myths surrounding Sigmund Freud’s dream theory: a reply to Hobson’s critique of the scientific status of psychoanalysis.. *Contemp. Psychoanal.*.

[B21] Compton A. (1972). A study of the psychoanalytic theory of anxiety: I. The development of Freud’s theory of anxiety. *J. Am. Psychoanal. Assoc.* 20 3–44 10.1177/000306517202000101

[B22] Compton A. (1981). On the psychoanalytic theory of instinctual drives. IV: instinctual drives and the ego-id-superego model. *Psychoanal. Q.* 50 363–392 7267867

[B23] Dalenberg C. J. Brand B. L. Gleaves D. H. Dorahy M. J. Loewenstein R. J. Cardeña E. (2012). Evaluation of the evidence for the trauma and fantasy models of dissociation. *Psychol. Bull.* 138 550–588 10.1037/a0027447 22409505

[B24] Dalenberg C. J. Brand B. L. Loewenstein R. J. Gleaves D. H. Dorahy M. J. Cardeña E. (2014). Reality versus fantasy: reply to Lynn et al.(2014). *Psychol. Bull.* 140 911–920 10.1037/a0036685 24773506

[B25] Dreher A. U. (2005). “Conceptual research,” in *Textbook of Psychoanalysis* edsPerson E. S. Cooper A. M. Gabbard G. O. (Washington, DC: American Psychiatric Publishing) 361–372

[B26] Erwin E. (1988). “Psychoanalysis & self-deception,” in *Perspectives on Self-deception* edsMcLaughlin B. P. Rorty A. O. (Berkeley: University of California Press) 228–245

[B27] Fairbairn W. R. D. (1931/1952). “Features in the analysis of a patient with a physical genital abnormality,” in *Psychoanalytic Studies of the Personality* (London: Tavistock) 197–222

[B28] Fairbairn W. R. D. (1943/1952). “The repression and the return of bad objects with special reference to the ‘War Neuroses,”’ in *Psychoanalytic Studies of the Personality* (London: Tavistock) 59–81

[B29] Fairbairn W. R. D. (1944/1952). “Endopsychic structure considered in terms of object-relationships,” in *Psychoanalytic Studies of the Personality* (London: Tavistock) 82–136

[B30] Fairbairn W. R. D. (1951/1952). “A synopsis of the development of the author’s views regarding the structure of personality,” in *Psychoanalytic Studies of the Personality* (London: Tavistock) 162–179

[B31] Fairbairn W. R. D. (1952). *Psychoanalytic Studies of the Personality*. London: Tavistock

[B32] Freud A. (1968). *The Ego and the Mechanisms of Defence*. London: The Hogarth Press

[B33] Freud S. (1895). *Project for a Scientific Psychology* Standard Edn Vol. I. London: Hogarth

[B34] Freud S. (1900). *The Interpretation of Dreams* Standard Edn Vols. IV and V. London: Hogarth

[B35] Freud S. (1905a). *Fragment of an Analysis of a Case of Hysteria* Standard Edn Vol. VII. London: Hogarth

[B36] Freud S. (1905b).*Three Essays on the Theory of Sexuality* Standard Edn Vol. VII. London: Hogarth

[B37] Freud S. (1907). *Delusions and Dreams in Jensen’s Gradiva* Standard Edn Vol. IX. London: Hogarth

[B38] Freud S. (1910). *The Psycho-Analytic View of the Psychogenic Disturbance of Vision* Standard Edn Vol. XI. London: Hogarth

[B39] Freud S. (1911a). *Psycho-Analytic Notes on an Autobiographical Account of a Case of Paranoia (Dementia Paranoides)* Standard Edn Vol. XII. London: Hogarth

[B40] Freud S. (1911b). *Formulations on the Two Principles of Mental Functioning* Standard Edn Vol. XII. London: Hogarth

[B41] Freud S. (1912). *On the Universal Tendency to Debasement in the Sphere of Love (Contributions to the Psychology of Love II)* Standard Edn Vol. XI. London: Hogarth

[B42] Freud S. (1914). *On Narcissism: An Introduction* Standard Edn Vol. XIV. London: Hogarth

[B43] Freud S. (1915a). *Instincts and their Vicissitudes* Standard Edn Vol. XIV. London: Hogarth

[B44] Freud S. (1915b). *Repression* Standard Edn Vol. XIV London: Hogarth

[B45] Freud S. (1915c). *The Unconscious* Standard Edn Vol. XIV. London: Hogarth

[B46] Freud S. (1920). *Beyond the Pleasure Principle* Standard Edn Vol. XVIII London: Hogarth

[B47] Freud S. (1923a). *Two Encyclopaedia Articles* Standard Edn Vol. XVIII London: Hogarth

[B48] Freud S. (1923b). *The Ego and the Id* Standard Edn Vol. XIX. London: Hogarth

[B49] Freud S. (1924). *The Economic Problem of Masochism* Standard Edn Vol. XIX. London: Hogarth

[B50] Freud S. (1925a). *Some Additional Notes on Dream-Interpretation as a Whole* Standard Edn Vol. XIX. London: Hogarth

[B51] Freud S. (1925b). *The Resistances to Psycho-Analysis* Standard Edn Vol. XIX. London: Hogarth

[B52] Freud S. (1925c). *An Autobiographical Study* Standard Edn Vol. XX. London: Hogarth

[B53] Freud S. (1926). *Inhibitions, Symptoms and Anxiety* Standard Edn Vol. XX. London: Hogarth

[B54] Freud S. (1930). *Civilization and its Discontents* Standard Edn Vol. XXI. London: Hogarth

[B55] Freud S. (1933). *New Introductory Lectures on Psycho-Analysis* Standard Edn Vol. XXII. London: Hogarth

[B56] Freud S. (1935). *The Subtleties of a Faulty Action* Standard Edition Vol. XXII. London: Hogarth

[B57] Freud S. (1939). *Moses and Monotheism: Three Essays* Standard Edn Vol. XXIII. London: Hogarth

[B58] Freud S. (1940). *An Outline of Psycho-Analysis* Standard Edn Vol. XXIII. London: Hogarth

[B59] Fulgencio L. (2005). Freud’s metapsychological speculations. *Int. J. Psychoanal,* 86 99–123 10.1516/TNY1-6YT7-V37T-16LD 15859224

[B60] Gardner S. (1993). *Irrationality and the Philosophy of Psychoanalysis*. Cambridge: Cambridge University Press.doi: 10.1017/CBO9780511554599

[B61] Gill M. M. (1963). *Topography and Systems in Psychoanalytic Theory*. New York: International Universities Press.doi: 10.1037/13109-000

[B62] Gillett E. (1997). Revising Freud’s structural theory. *Psychoanal. Contemp. Thought* 20 471–499

[B63] Green A. (2005). The illusion of common ground and mythical pluralism. *Int. J. Psychoanal.* 86 627–632 16106577

[B64] Greenberg J. R. Mitchell S. A. (1983). *Object Relations in Psychoanalytic Theory*. Cambridge, MA: Harvard University Press

[B65] Grossman W. I. (1982). The self as fantasy: fantasy as theory. *J. Am. Psychoanal. Assoc.* 30 919–937 10.1177/000306518203000405 6927245

[B66] Grossman W. I. Simon B. (1969). Anthropomorphism: motive, meaning, and causality in psychoanalytic theory. *Psychoanal. Study Child* 24 78–111 5353376

[B67] Guntrip H. (1968). *Personality Structure and Human Interaction*. London: Hogarth

[B68] Hartmann H. (1950). Comments on the psychoanalytic theory of the ego. *Psychoanal. Study Child* 5 74–96

[B69] Hartmann H. (1958). *Ego Psychology and the Problem of Adaptation*. New York: International Universities Press.doi: 10.1037/13180-000

[B70] Holt R. R. (1976). “Drive or wish? a reconsideration of the psycho-analytic theory of motivation,” in *Psychology vs Metapsychology: Psycho-Analytic Essays in Memory of George Klein, Psychological issues Monograph 36,* Vol. 9 edsGill M. Holzman P. (New York, NY: International Universities Press) 158–197

[B71] James W. (1890/1950). *The Principles of Psychology* Vol. 1. New York: Henry Holt and Company

[B72] Kernberg O. (2009). The concept of the death drive: a clinical perspective. *Int. J. Psychoanal.* 90 1009–1023 10.1111/j.1745-8315.2009.00187.x 19821849

[B73] Khantzian E. J. Mack J. E. (1983). Self-preservation and the care of the self: ego instincts reconsidered. *Psychoanal. Study Child* 38 209–232 6647652 10.1080/00797308.1983.11823390

[B74] Kluft R. P. (2000). The psychoanalytic psychotherapy of dissociative identity disorder in the context of trauma therapy. *Psychoanal. Inquiry* 20 259–286 10.1080/07351692009348887

[B75] Laplanche J. Pontalis J.-B. (1973). *The Language of Psychoanalysis*. London: Karnac

[B76] Lettieri R. (2005). The ego revisited. *Psychoanal. Psychol*. 22 370–381 10.1037/0736-9735.22.3.370

[B77] Leuzinger-Bohleber M. Fischmann T. (2006). What is conceptual research in psychoanalysis. *Int. J. Psychoanal.* 87 1355–1386 10.1516/73MU-E53N-D1EE-1Q8L 16997730

[B78] Lynn S. J. Lilienfeld S. O. Merckelbach H. Giesbrecht T. McNally R. J. Loftus E. F. (2014). The trauma model of dissociation: inconvenient truths and stubborn fictions. Comment on Dalenberg etal (2012). *Psychol. Bull.* 140 896–910 10.1037/a0035570 24773505

[B79] Marcus E. R. (1999). Modern ego psychology. *J. Am. Psychoanal. Assoc.* 47 843–871 10.1177/00030651990470031501 10586403

[B80] Madison P. (1956). Freud’s repression concept: a survey and attempted clarification. *Int. J. Psychoanal.* 37 75–81 13318727

[B81] Maze J. R. (1983). *The Meaning of Behaviour*. London: Allen & Unwin

[B82] Maze J. R. (1987). “The composition of the ego in a deterministic psychology,” in *Current Issues in Theoretical Psychology* edsBaker W. J. Hyland M. E. Van Rappard H. Staats A. W. (North Holland: Elsevier Science Publishers) 189–199

[B83] Maze J. R. (1993). The complementarity of object-relations and instinct theory. *Int. J. Psychoanal.* 74 459–470 8344767

[B84] Maze J. R. Henry R. M. (1996). Problems in the concept of repression and proposals for their resolution. *Int. J. Psychoanal.* 77 1085–1100 9119578

[B85] McEwen B. S. Wingfield J. C. (2010). What is in a name? Integrating allostasis and stress. *Horm. Behav.* 57 105–111 10.1016/j.yhbeh.2009.09.011 19786032 PMC2815096

[B86] McIlwain D. (2007). Rezoning pleasure: drives and affects in personality theory. *Theory Psychol.* 17 529–561 10.1177/0959354307079303

[B87] Meissner W. W. (2007). Therapeutic alliance: theme and variations. *Psychoanal. Psychol.* 24 231–254 10.1037/0736-9735.24.2.231

[B88] Newbery G. (2011). “Drive theory reconsidered (again!),” in *Realism and Psychology: Collected Essays* edsMackay N. Petocz A. (Leiden: Brill) 839–871

[B89] Ogden T. H. (1993). *The Matrix of the Mind: Object Relations and the Psychoanalytic Dialogue*. London: Karnac

[B90] Ogden T. H. (2002). A new reading of the origins of object-relations theory. *Int. J. Psychoanal.* 83 767–782 10.1516/LX9C-R1P9-F1BV-2L96 12204163

[B91] Ogden T. H. (2010). Why read Fairbairn? *Int. J. Psychoanal*. 91 101–118 10.1111/j.1745-8315.2009.00219.x 20433477

[B92] Ogden T. H. (2011). Reading Susan Isaacs: towards a radically revised theory of thinking. *Int. J. Psychoanal.* 92 925–942 10.1111/j.1745-8315.2011.00413.x 21843242

[B93] Panksepp J. (1998). *Affective Neuroscience: The Foundations of Human and Animal Emotions*. New York: Oxford

[B94] Panksepp J. Moskal J. (2008). “Dopamine and SEEKING: subcortical “reward” systems and appetitive urges,” in *Handbook of Approach and Avoidance Motivation* ed.Elliot A. J. (New York: Psychology Press) 67–87

[B95] Passmore J. A. (1935). The nature of intelligence. *Aust. J. Psychol. Philos.* 13 279–289

[B96] Pataki T. (2014). “Fairbairn and partitive conceptions of mind,” in *Fairbairn and the Object Relations Tradition* edsScharff D. Clarke G. (London: Karnac) 417–430

[B97] Petocz A. (1999). *Freud, Psychoanalysis, and Symbolism*. Cambridge: Cambridge University Press.doi: 10.1017/CBO9780511583452

[B98] Piper A. Merskey H. (2004a). The persistence of folly: a critical examination of dissociative identity disorder. Part I. The excesses of an improbable concept. *Can. J. Psychiatry* 49 592–600 15503730 10.1177/070674370404900904

[B99] Piper A. Merskey H. (2004b). The persistence of folly: critical examination of dissociative identity disorder. Part II. The defence and decline of multiple personality or dissociative identity disorder. *Can. J. Psychiatry* 49 678–683 15560314 10.1177/070674370404901005

[B100] Plato (1928). *The Republic (introduction by C. M. Bakewell)*. New York: Charles Schribner’s Sons

[B101] Pulcu E. (2014). An evolutionary perspective on gradual formation of superego in the primal horde. *Front. Psychol.* 5:8 10.3389/fpsyg.2014.00008 PMC3900855 24478740

[B102] Rapaport D. (1951). “Toward a theory of thinking,” in *Organization and Pathology of Thought: Selected Sources* ed.Rapaport D. (New York: Columbia University Press) 689–730

[B103] Reinders A. S. Willemsen A. T. Vos H. P. den Boer J. A. Nijenhuis E. R. (2012). Fact or factitious? A psychobiological study of authentic and simulated dissociative identity states. *PLoS ONE* 7:e39279 10.1371/journal.pone.0039279 PMC3387157 22768068

[B104] Ritvo S. Solnit A. J. (1995). “Instinct theory,” in *Psycho-analysis: The Major Concepts* edsMoore B. E. Fine B. D. (New Haven: Yale University Press) 327–333

[B105] Rosenblatt A. D. Thickstun J. T. (1977). Energy, information, and motivation: a revision of psycho-analytic theory. *J. Am. Psychoanal. Assoc.* 25 537–558 10.1177/000306517702500302 915197

[B106] Schlumpf Y. R. Nijenhuis E. R. Chalavi S. Weder E. V. Zimmermann E. Luechinger R. (2013). Dissociative part-dependent biopsychosocial reactions to backward masked angry and neutral faces: an fMRI study of dissociative identity disorder. *Neuroimage* 3 54–64 10.1016/j.nicl.2013.07.002 24179849 PMC3791283

[B107] Schulkin J. (2003). *Rethinking Homeostasis: Allostatic Regulation in Physiology and Pathophysiology*. Cambridge, MA: MIT Press

[B108] Segal H. (1993). On the clinical usefulness of the concept of death instinct. *Int. J. Psychoanal.* 74 55–61 8454404

[B109] Sewards T. V. Sewards M. A. (2002). The medial pain system: neural representations of the motivational aspects of pain. *Brain Res. Bull.* 59 163–180 10.1016/S0361-9230(02)00864-X 12431746

[B110] Sewards T. V. Sewards M. A. (2003). Representations of motivational drives in mesial cortex, medial thalamus, hypothalamus and midbrain. *Brain Res. Bull.* 61 25–40 10.1016/S0361-9230(03)00069-8 12788205

[B111] Shill M. A. (2004). Signal anxiety, defense, and the pleasure principle. *Psychoanal. Psychol.* 21 116–133 10.1037/0736-9735.21.1.116

[B112] Slap J. W. Saykin A. J. (1984). On the nature and the organisation of the repressed. *Psychoanal. Inquiry* 4 107–124 10.1080/07351698409533533

[B113] Slavin M. O. Grief D. (1995). “The evolved function of repression and the adaptive design of the human psyche,” in *Ego Defenses: Theory and Measurement* edsConte H. R. Plutchik R. (New York: John Wiley & Sons) 139–175

[B114] Solms M. Panksepp J. (2012). The “id” knows more than the “ego” admits: neuropsychoanalytic and primal consciousness perspectives on the interface between affective and cognitive neuroscience. *Brain Sci.* 2 147–175 10.3390/brainsci2020147 24962770 PMC4061793

[B115] Symington N. (1993). *Narcissism: A New Theory*. London: Karnac

[B116] Talvitie V. (2012). *The Foundations of Psychoanalytic Theories: Project for a Scientific Enough Psychoanalysis*. London: Karnac

[B117] Tauber A. I. (2010). *Freud, the Reluctant Philosopher*. Princeton: Princeton University Press

[B118] Wagner H. (1999). *The Psychobiology of Human Motivation*. London: Routledge

[B119] Wallerstein R. S. (2005a). Will psychoanalytic pluralism be an enduring state of our discipline? *Int. J. Psychoanal*. 86 623–626 10.1516/VJHB-7R8A-162R-B5WN 16096067

[B120] Wallerstein R. S. (2005b). Dialogue or illusion? How do we go from here? Response to André Green. *Int. J. Psychoanal.* 86 633–638

[B121] Westen D. (1997). Towards a clinically and empirically sound theory of motivation. *Int. J. Psychoanal.* 78 521–548 9257166

[B122] Wiedeman G. H. (1972). Comments on the structural theory of personality. *Int. J Psychoanal.* 53 307–313 5057077

[B123] Wollheim R. (1991). *Freud*. London: Fontana Press

[B124] Young-Bruehl E. Bethelard F. (1999). The hidden history of the ego instincts. *Psychoanal. Rev*. 86 823–851

